# Patterns of taxonomic diversity among terrestrial isopods

**DOI:** 10.3897/zookeys.515.9332

**Published:** 2015-07-30

**Authors:** Spyros Sfenthourakis, Stefano Taiti

**Affiliations:** 1 University of Cyprus, Department of Biological Sciences, P.O. Box 20537, 1678 Nicosia, Cyprus; 2 Istituto per lo Studio degli Ecosistemi, Consiglio Nazionale delle Ricerche, Via Madonna del Piano 10, I-50019 Sesto Fiorentino, Florence, Italy

**Keywords:** Bodiversity, diversification, systematics, fractals, phylogeny, species richness, taxonomic asymmetry

## Abstract

The publication of the world catalog of terrestrial isopods some ten years ago by Schmalfuss has facilitated research on isopod diversity patterns at a global scale. Furthermore, even though we still lack a comprehensive and robust phylogeny of Oniscidea, we do have some useful approaches to phylogenetic relationships among major clades which can offer additional insights into isopod evolutionary dynamics. Taxonomic diversity is one of many approaches to biodiversity and, despite its sensitiveness to biases in taxonomic practice, has proved useful in exploring diversification dynamics of various taxa. In the present work, we attempt an analysis of taxonomic diversity patterns among Oniscidea based on an updated world list of species containing 3,710 species belonging to 527 genera and 37 families (data till April 2014). The analysis explores species diversity at the genus and family level, as well as the relationships between species per genera, species per families, and genera per families. In addition, we consider the structure of isopod taxonomic system under the fractal perspective that has been proposed as a measure of a taxon’s diversification. Finally, we check whether there is any phylogenetic signal behind taxonomic diversity patterns. The results can be useful in a more detailed elaboration of Oniscidea systematics.

## Introduction

Terrestrial isopods constitute one of the most remarkable lineages of invertebrates that managed to conquer land. Modern species represent almost all evolutionary steps that enabled them to leave the marine environment and occupy almost the whole range of terrestrial habitat types. This makes them a unique case within global biodiversity and offers lots of opportunities to biological research, especially in fields like evolution, ecology and ecophysiology (Warburg 1993). Even though the phylogeny of terrestrial isopods has not yet been adequately resolved, the monophyly of certain large clades is well supported, and we do have a relatively good picture of the major evolutionary transitions that made possible the conquest of more and more arid habitat types.

Terrestrial isopods, the suborder Oniscidea within the order Isopoda, are currently considered to be a monophyletic taxon even though their monophyletic origin has been questioned in the past (see Schmidt 2008 for a review). The status of the family Tylidae might still be considered somewhat ambiguous but, in general, most authors agree that isopods invaded land from marine ancestors, most probably once in their history. They have evolved a number of unique adaptations with no parallels in other related taxa (see Hornung 2011 for a review), such as the water conducting system, the various forms of pleopodal lungs and the cotyledons in the marsupium. In fact, the last two structures might be considered as analogous to the lungs in vertebrates and the placenta in mammals, respectively.

From an ecological point of view, Oniscidea live in almost all biomes, having successfully invaded most areas of the world, with the exception of the poles and very high elevations (>4,800 m, Beron 1997). In some ecosystems they constitute one of the most important components of decomposer communities, being largely phytosaprophagous and often occurring at very high population densities (Dias and Hassall 2005). Some species that live in very harsh desert environments have even attained the level of subsocial organization. In addition, terrestrial isopods are amongst the most common and species-rich components of cave-dwelling animal groups with very large percentages of troglobitic species. Oniscidea also include amphibious and even aquatic species that have secondarily returned to live in salt lakes or subterranean freshwaters (Tabacaru 1999, Taiti and Xue 2012).

Oniscidea probably originated in the Carboniferous (Broly et al. 2013), and are represented today by more than 3,700 species belonging to more than 500 genera in 37 families and five higher clades (infraorders/sections) (Schmidt 2008). The long evolutionary history of terrestrial isopods has led to considerably asymmetric species-richness patterns among major clades. The more basal Diplocheta and Tylida are relatively species-poor, Mesoniscidae are represented by just two species, while the vast majority of species belong to the, more apical, sister clades Synocheta and Crinocheta, with the latter being more ‘terrestrial’ and by far the richest in species number. Such asymmetries in taxonomic richness among clades might reflect differences in evolutionary dynamics, such as the relative strength of evolvability and genetic constraints, or the roles of key innovations in the rates of lineage diversification. Therefore, the identification of significant patterns in species richness within clades can provide important insights into the history of biodiversity.

The study of taxonomic diversity aims to explore such patterns across different taxonomic levels. Despite its sensitiveness to biases in taxonomic practice it has proved useful in exploring diversification dynamics in characteristic biota (e.g., Simberloff 1970), plant (Väre et al. 2003) or animal groups (Larson et al. 2001, Sierwald and Bond 2007, Pincheira-Donoso et al. 2013). Obviously, exploration of taxonomic diversity should be based on a comprehensive account of the species known within the respective lineage. The publication of the world catalog of terrestrial isopods some ten years ago (Schmalfuss 2003, 2004) has facilitated this line of research on isopod diversity patterns at a global scale, even though the isopod fauna of large parts of the world remains largely unexplored or, at least, inadequately known. Nevertheless, our current knowledge on the group can provide a relatively solid basis for a preliminary analysis of its taxonomic diversity that, in turn, might identify important gaps and other issues that should come into the focus of future research. Such an analysis can also contribute to the broader discussion on patterns of diversification rates, such as the possible tendency for exceptionally rich clades to be rare or the fractal structure of taxonomic levels (Burlando 1990).

In the present work we attempt an analysis of taxonomic diversity patterns among Oniscidea based on an updated world list of species, exploring species diversity at the genus and family level, as well as the relationships between species per genera, species per families, and genera per families. Even though the assignment of genus and family status for a group of species or clades is arbitrary, experts in each higher taxon usually follow a similar approach, so that the study of such patterns is still meaningful to a considerable extent. Also, we do know that several families (or even genera) of Oniscidea might not be monophyletic, and this could lead to uncertainties in results from such a taxonomic diversity analysis. Nevertheless, this kind of analysis actually helps towards identifying such problems. For example, the exploration of a possible fractal structure in the isopod taxonomic system, which has been proposed as a measure of diversification, is a useful tool for this, and we do address the issue herein. Finally, we check whether there is any phylogenetic signal behind taxonomic diversity patterns.

## Methods

In order to compile a complete species list of valid isopod species, we used as a basis the world catalog published by Schmalfuss (2003) and its updated electronic version (Schmalfuss 2004). To this we added all taxonomic changes made till April 2014 (new species descriptions plus nomenclatural changes). For nomenclature and familiar assignments, we followed Schmidt (2008). Some problematic cases were identified as such and left outside further treatment. The final complete species list per family and genus was compiled after a thorough evaluation of each species’ known status by the second author (ST). The complete list is available by the authors upon request.

Species description rates were calculated from dates appearing in current nomenclature.

For phylogenetic information, we used the morphological analysis by Erhard (1998) and Schmidt (2008), and the molecular analysis by Mattern and Schlegel (2001).

In order to test for skewness in the frequency distribution of species richness at different taxonomic levels we used the standardized skewness metric and the Shapiro Wilk test for deviation from normality. In all other analyses we applied standard linear regressions and the Pearson product moment, or the Spearman rank correlation coefficient.

## Results

In the ten years after the publication of the electronic version of the world catalog of terrestrial isopod species (Schmalfuss 2004), less than 80 new species were added. In particular, 3,637 species were included in the 2004 list, while 3,710 species are recognized in our 2014 (as of April) compilation. These species belong to 527 genera and 37 families. The complete list includes 192 species of ambiguous generic assignment and 37 genera (that include 90 species overall) of ambiguous familial assignment.

The list of families with the respective numbers of genera and species is given in Table 1.

**Table 1.** List of families with their respective numbers of genera and species, the latter separately for those in known genera and those of uncertain generic assignment.

Family	Number of genera	Species in known genera	Species of uncertain generic assignment	Total
Armadillidae	80	579	118	**697**
Philosciidae	107	501	36	**537**
Trichoniscidae	87	492	2	**494**
Porcellionidae	19	326	7	**333**
Armadillidiidae	14	256	0	**256**
Eubelidae	50	253	2	**255**
Agnaridae	14	157	10	**167**
Platyarthridae	7	122	0	**122**
Trachelipodidae	6	109	4	**113**
Scleropactidae	26	107	0	**107**
Ligiidae	6	95	0	**95**
Styloniscidae	10	81	1	**82**
Cylisticidae	5	66	0	**66**
Detonidae	4	39	0	**39**
Halophilosciidae	3	35	0	**35**
Oniscidae	5	31	10	**41**
Alloniscidae	2	23	2	**25**
Tylidae	2	22	0	**22**
Spelaeoniscidae	7	20	0	**20**
Delatorreidae	3	18	0	**18**
Dubioniscidae	3	15	0	**15**
Rhyscotidae	2	13	0	**13**
Olibrinidae	4	11	0	**11**
Scyphacidae	2	11	0	**11**
Bathytropidae	1	10	0	**10**
Balloniscidae	2	8	0	**8**
Titaniidae	5	6	0	**6**
Tendosphaeridae	3	4	0	**4**
Stenoniscidae	2	4	0	**4**
Pudeoniscidae	2	4	0	**4**
Irmaosidae	1	2	0	**2**
Mesoniscidae	1	2	0	**2**
Schoebliidae	1	2	0	**2**
Berytoniscidae	1	1	0	**1**
Bisilvestriidae	1	1	0	**1**
Hekelidae	1	1	0	**1**
Turanoniscidae	1	1	0	**1**
Unknown	37	90	0	**90**
Total	527	3,518	192	**3,710**

The family with the highest species richness is Armadillidae, followed by Philosciidae and Trichoniscidae. The same three families are also the richest in genera, albeit in a different order, with Philosciidae first, followed by Trichoniscidae and Armadillidae. There are seven monogeneric families, four of them with monotypic genera. Species richness is significantly correlated with genera richness (Spearman rank correlation coefficient: *r_s_* = 0.91, *p* < 0.001).

**Figure 1.**
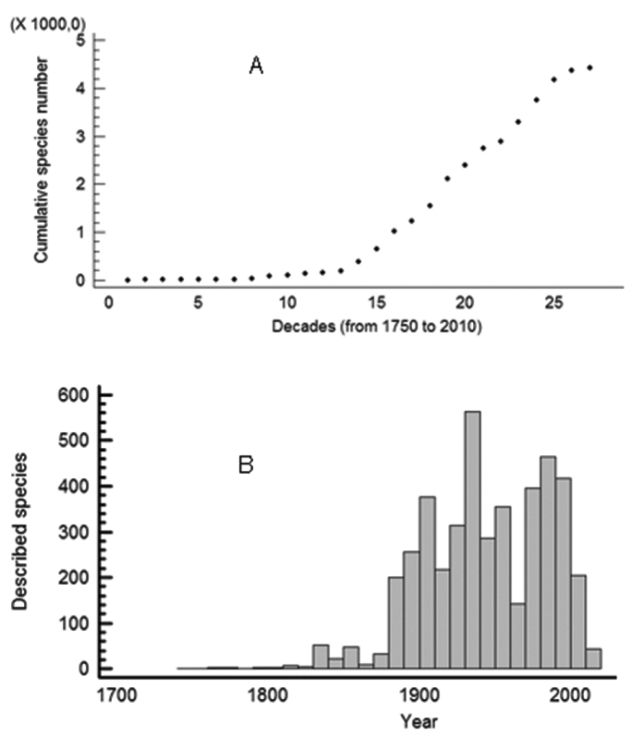
Rate of isopod species description since 1750. **A** Number of terrestrial isopod species described per decade **B** Cumulative species number of terrestrial isopods per decade, since 1750.

Species descriptions per decade showed a bimodal distribution in the last century with most of the currently valid species being described either in the first half of the 20^th^ century, especially in the ‘20s, or from 1960 to 1990 (Fig. 1A). The cumulative number of species seems to have reached a plateau in the last two decades (Fig. 1A).

**Figure 2.**
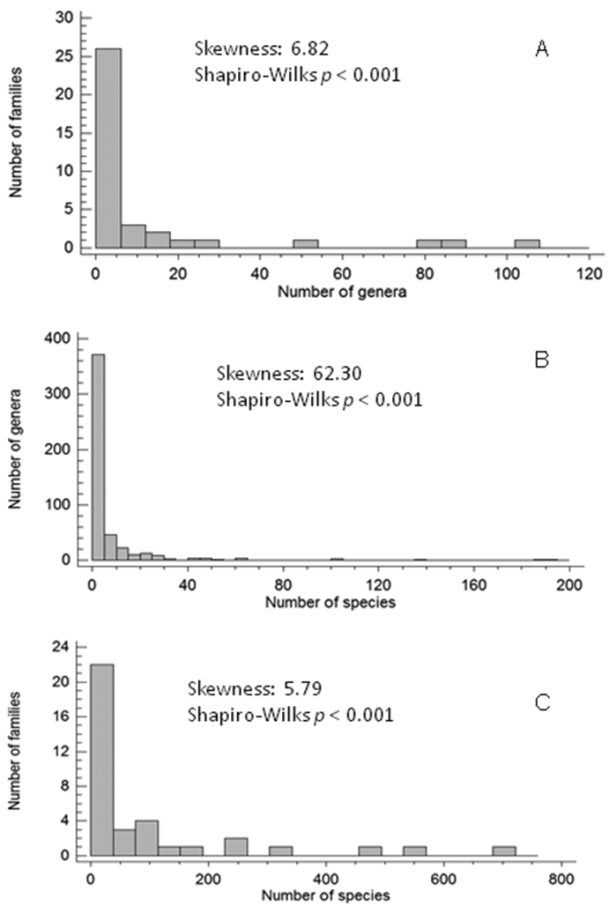
Skewness in the distribution of taxonomic richness with results of the respective Shapiro-Wilks tests. **A** for number of genera per families **B** for number of species per genera, and **C** for number of species per families.

Frequency distributions of isopod richness are significantly right-skewed (for genera within families: skewness = 6.82, Shapiro-Wilk test *p* < 0.001; for species within genera: skewness = 62.3, Shapiro-Wilk test *p* < 0.001; for species within families: skewness = 5.7, Shapiro-Wilk test *p* < 0.001). This means that most families and genera consist of few genera and species, respectively, while very rich lineages are rare (Fig. 2).

**Figure 3.**
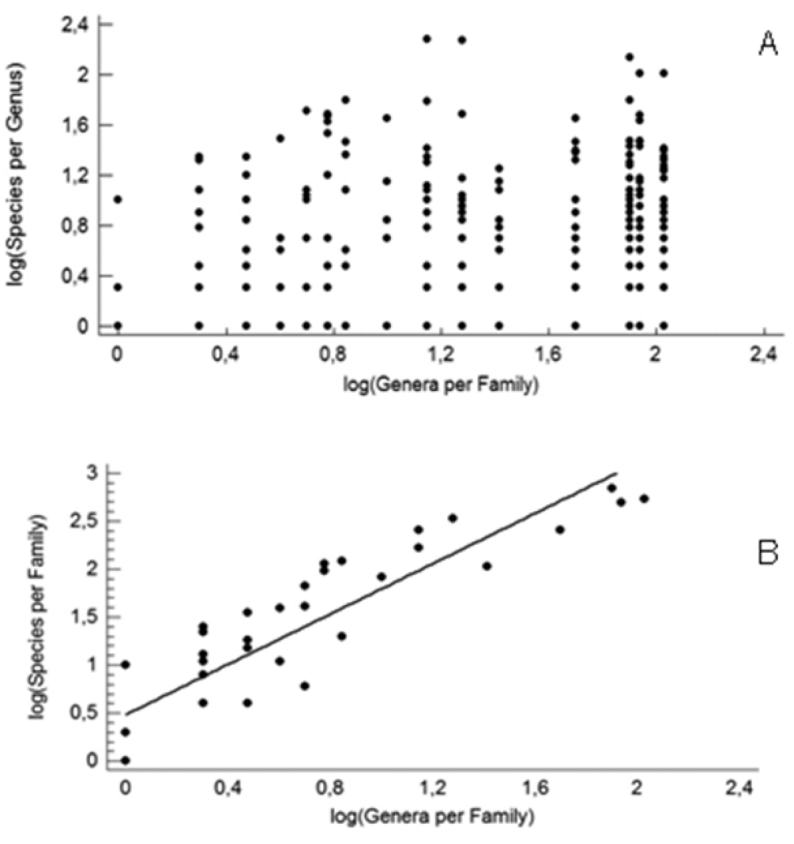
Regression of species richness per higher taxonomic groups against richness of genera per families (all in logarithmic values). **A** species per genera **B** species per family.

The number of species per genus in a family is not predicted by the number of genera per family (Fig. 3A). On the other hand, the number of species per family is positively correlated with the number of genera per family (*r* = 0.90, *p* < 0.001; Fig. 3B).

**Figure 4.**
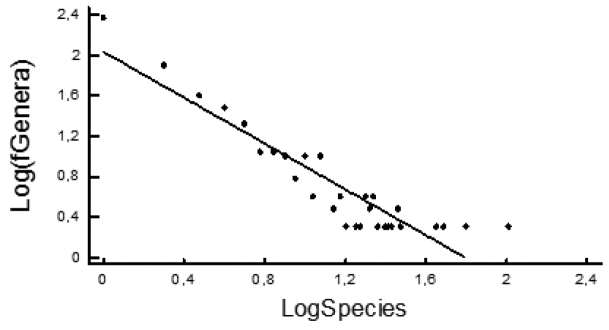
Linear regression of the frequency of genera (fGenera) against their respective species richness, revealing the fractal nature of terrestrial isopod taxonomy. The slope of the regression (1.14) gives the fractal dimension. Unit frequency values – genera with unique values of species richness – have been excluded in order to avoid a long queue of zeros that smoothens the slope only due to the fact that the size of large genera is more probable to be unique.

The frequency of genera is negatively correlated with the number of species per genus (in logarithmic space: *r* = -0.88, *p* < 0.001; Fig. 4), giving a fractal dimension (= the absolute value of the slope of the respective linear regression) of 1.02, which becomes 1.14 when unit values are excluded from the analysis (to avoid the long tail of zeros, i.e., very large genera).

**Figure 5.**
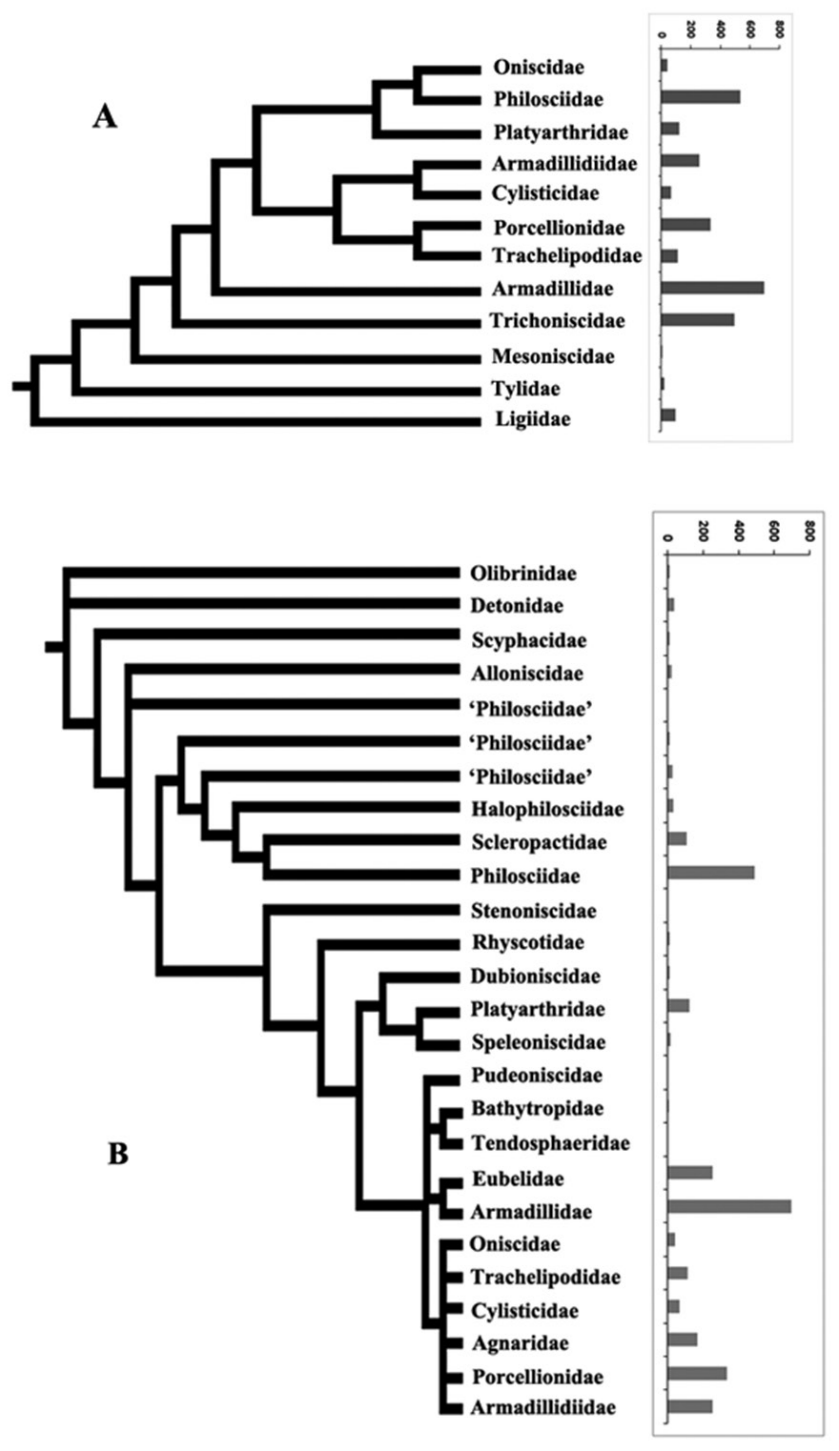
The species richness of Oniscidea families against two phylogenetic hypotheses. **A** Tree from Mattern and Schlegel (2001) incorporating also hypotheses from Erhard (1998) **B** tree for Crinocheta from Schmidt (2008).

Species richness values per family were mapped on the available phylogenetic trees for Oniscidea (Fig. 5A, B) to see whether there is any apparent phylogenetic signal in richness patterns. It is obvious that basal clades are poor, but inside the more derived clade Crinocheta-Synocheta the picture is not very clear (Fig. 5A). Nevertheless, inside Crinocheta (Fig. 5B) it seems that species richness is much higher in the more derived clades.

## Discussion

Terrestrial isopods are the largest suborder of Isopoda and actually the only group of Crustacea that has managed to exploit almost the whole range of terrestrial ecosystems. The ca. 3,700 species known so far include clades that have evolved a variety of morphological, physiological and behavioral characters offering unique solutions to key problems pertaining to the adaptation to the life on land, so that today they represent almost all transitional stages from marine to extremely arid environments.

According to the rate of species descriptions presented herein one might assume that the vast bulk of the global oniscid diversity has been known, and the total richness will not change to a significant degree in the near future. Nevertheless, we should note that the ‘plateau’ in the accumulated species richness observed in the last two decades might be better attributed to the decline in taxonomic expertise on the group. Indeed, there are very few active taxonomists of Oniscidea today. A large part of the world remains unexplored, especially the tropics, and the current trends in funding and ‘academic prestige’ do not leave much space for optimism that they will be explored soon. It is equally important to note that many thousands of caves around the world are expected to host hundreds, if not thousands, of isopod species, taking into account that Oniscidea are amongst the richest animal taxa in troglobitic species, most of which occur in one or a few local caves and/or other subterranean habitats. Furthermore, several analyses based on molecular markers reveal an even higher diversity among isopod taxa (e.g., Klossa-Kilia et al. 2006, Parmakelis et al. 2008, Hurtado et al. 2010, 2013, Kamilari et al. 2014). With the increasing application of molecular techniques in isopod phylogeny and taxonomy, we expect that more and more ‘cryptic’ species will be found. A reasonable estimation of the expected global richness of Oniscidea might be between 5,000 and 7,000 species. From a biogeographical perspective, and despite the reasonable bias in Europe due to the geography of research and researchers, it still remains true that the Mediterranean region is particularly rich in Oniscidea and the same is true also for other areas of the world with Mediterranean-type ecosystems, such as South Africa and western Australia. Furthermore, terrestrial isopods are highly diversified also in tropical regions, especially in areas with increased environmental heterogeneity. We still lack a concise biogeographical analysis of Oniscidea at a global scale, but all evidence from narrower geographical scales show that isopod richness is highly correlated with geographical and landscape complexity (e.g., presence of islands, cave systems, mountainous regions) and environmental heterogeneity (e.g., Mediterranean).

The analysis of global diversity conducted herein reveals a strong right-skewed frequency distribution, so that Oniscidea mostly contain genera with few species and families with few genera. This is a pattern observed also in other animal taxa (e.g., hexapoda: Mayhew 2001, mammals: Purvis et al. 2011, reptiles: Pincheira-Donoso et al. 2013) suggesting that even though very diverse lineages are rare, they contribute significantly to the total diversity of each higher taxon. In fact, patterns of taxonomic diversity identified for Oniscidea show more similarities with those in other organisms, suggesting that taxonomic structure might not be idiosyncratic for each higher taxon. This might be related to the critical role of key innovations in clade diversification, such as improved resistence towards dehydration that enabled Crinocheta to conquer new ‘macro-niches’, such as habitats with less relative humidity, more arid habitats etc. (see Schmalfuss 1998).

The correlation between number of species and number of genera in a family, in combination with the fact that numbers of species per genus cannot predict generic richness in a family, underlines the wide variation inside Oniscidea. This is because in addition to the somewhat trivial fact that many small genera – even monotypic – may be found in large families, the pattern is also based on the occurrence of very diverse genera in small families.

If fractal geometry of taxonomic systems indeed reflects real patterns of evolutionary diversification, then isopod diversity appears underestimated. The ‘fractal dimension’ of most arthropods and North American isopods (including freshwater and marine) is 1.50 according to Burlando (1990), while for Aegean terrestrial isopods it is 1.48 (Sfenthourakis 1994). Lower values suggest either lower diversification or the need for further splitting of large taxa. Given that diversification cannot be considered low in Oniscidea, the 1.14 value found herein implies that large genera might not be monophyletic units and need to be split into smaller ones. The possible non-monophyly of many higher taxa inside Oniscidea is a suggestion regularly reported in the relevant literature, and recent molecular analyses seem to also support such statements (e.g., *Hemilepistus*: Dimitriou, Kashani and Sfenthourakis, in preparation).

An intriguing question refers to the role of ‘key innovations’ in adaptive radiations, which would lead to prominent radiations in clades that have acquired some new feature offering significant selective advantages. Such a pattern would lead to very asymmetric phylogenies and key innovations could be mapped as defining synapomorphies of prolific clades. If the phylogeny of Schmidt (2008) proves to be correct, then such a case should be exemplified by the derived clade including the ‘highly terrestrial’ families Eubelidae, Armadillidae, Armadillidiidae, Porcellionidae, Trachelipodidae, Agnaridae, Cylisticidae and Oniscidae. On the other hand, the high diversity of Trichoniscidae and Philosciidae does not seem to conform to this pattern, unless some not currently obvious unique key innovation can be identified in these taxa in the future. An alternative explanation is that these families are not monophyletic, so the total richness of the actual monophyletic units produced from their split would be lower, but even then their richness would still be high enough to ask for an explanation.

It is absolutely necessary to have a robust phylogeny of Oniscidea families (and genera) in order to gain crucial insights into the evolution of this fascinating taxon. New techniques using Next Generation Sequencing can facilitate this task and provide very useful information.
